# Prediction of direct carbon emissions of Chinese provinces using artificial neural networks

**DOI:** 10.1371/journal.pone.0236685

**Published:** 2021-05-13

**Authors:** Hui Jin

**Affiliations:** School of Economics, Shanghai University of Finance and Economics, Shanghai, China; Qinghai University, CHINA

## Abstract

Closely connected to human carbon emissions, global climate change is affecting regional economic and social development, natural ecological environment, food security, water supply, and many other social aspects. In a word, climate change has become a vital issue of general concern in the current society. In this study, the carbon emission data of Chinese provinces in 1999–2019 are collected and analyzed, so as to identify the carbon emission of direct consumption per 10,000 residents in each province (including each municipal city and autonomous region) and the entire nation based on population data. The Arc Geographic Information Science Engine (ArcGIS Engine) and C#.NET platform are employed to call the MATLAB neural network toolbox. A model is selected and embedded in the prediction system to develop the entire system. This study demonstrates that the carbon emissions per resident in Northern China are significantly higher than those in Southern China, with the rate of carbon emissions continuing to increase over time. Compared with other models, the Elman neural network has a higher carbon emission prediction accuracy, but with more minor errors. For instance, its accuracy and prediction performance are improved by 55.93% and 19.48%, respectively, compared with the Backpropagation Neural Network (BPNN). The prediction results show that China is expected to reach its peak carbon emission in around 2025–2030. The above results are acquired based on the concept of carbon emissions and neural network model theories, supported by GIS component technology and intelligent methods. The feasibility of BPNN, Radial Basis Function (RBF) and Elman neural network models for predicting residential carbon emissions is analyzed. This study also designs a comprehensive, integrated and extensible visual intelligent platform, which is easy to implement and stable in operation. The trend and characteristics of carbon emission changes from 2027 to 2032 are explored and predicted based on the data about direct carbon emissions of Chinese provincial residents from 1999 to 2019, purposed to provide a scientific basis for the control and planning of carbon emissions.

## 1. Introduction

Over recent years, a series of environmental issues have increasingly endangered human beings’ living environment, including global warming, melting glaciers, rising sea levels, frequent extreme weathers, and desertification. Global climate change has also become an essential issue of the current society’s general concern [1]. As to the natural ecological environment, global warming has exacerbated the vulnerability of the natural ecological environment and led to frequent occurrences of extreme weathers, such as the El Niño phenomena, droughts and floods, changes in forest distribution patterns, rising sea levels, diminished biodiversity, and dwindled freshwater resources [2]. Global climate change is inextricably linked to the sustainable development of all countries across the world, which are actively exploring ways to curb further climate deterioration in the trend of global warming.

Carbon dioxide is the most important human-made greenhouse gas, and the sharp increase in its concentration is the primary factor for global warming [3]. All countries have implemented the strictest measures to cut carbon emissions [4]. As the largest developing country in the world today, China sees its energy consumption accounting for 21% of global energy consumption, becoming the country with the most massive energy consumption in the world. And its energy consumption is expected to occupy 25% of the global energy consumption by 2035. 2007 witnessed that China’s total carbon emissions surpassed that of the United States, ranked first in the world; then by 2012, China’s total carbon emissions reached the combined level of the United States and Europe [5]. As a large country, China actively undertakes emission reduction tasks based on its capabilities, while doing an excellent job in developing the national economy [6].

The carbon emissions from rapid urbanization and industrialization and from the direct consumption of residential energy account for 32.13%, becoming the majority of China’s greenhouse gas emissions [7]. Research has shown that in developed countries, such as the United States, as the industrial level continues to decrease, residential carbon emissions are concentrated in the electricity and natural gas; on the other hand, in China, residential carbon emissions are concentrated in coal and electricity. Natural gas and electricity have become two growth points for residential carbon emissions in China, where residents’ transportation carbon emissions also take a large share. However, Chinese residents’ transportation carbon emissions are only one-eighth of those in the United States [8]. Therefore, it is crucial to predict Chinese residents’ direct carbon emissions based on their current energy and carbon consumption levels in all provinces in China.

The neural network, a computer technology developed in recent years, relies on its unique network structure characteristics and data processing methods to deliver fruitful results in various fields [9]. Especially, using neural networks to build prediction models is a relatively common data processing method [10]. Many investigations have been made on the application of neural network models in prediction of carbon emissions. Ye et al. (2018) have used neural networks to predict carbon emissions in the construction industry, where the economic development and improvement of standards might significantly impact carbon dioxide emissions in the future [11]. Balki et al. (2018) have adopted backpropagation artificial neural networks to develop models that can estimate engine performance and exhaust emissions, finding that carbon emissions of ethanol vehicles are 6% lower than gasoline ones [12]. Liu et al. (2017) have combined the chaos theory with the Backpropagation Neural Network (BPNN) to fit and predict carbon emission time series without considering other factors, proposing a method that is easier and more accurate than other prediction methods [13]. Kim et al. (2017) have used the newly developed neural network model to make highly accurate prediction and effectively evaluate the carbon emissions across industries [14]. Therefore, the use of neural network models in carbon emissions prediction has become a common research topic in this field. However, most relevant works focus on automobiles, buildings and industries in developed countries, and few involve predictions of residential carbon emissions.

Due to their substantial application value, neural network models have achieved fruitful research results in many fields. Given there is strong nonlinear relationships among various factors that affect the direct carbon emissions of residents, neural network models can utilize their ability to approach nonlinear functions to predict direct residential carbon emissions in China’s provinces (municipalities). Neural network models have been applied in carbon emission prediction and analyses; however, these models have not been widely adopted in predicting the direct residential carbon emissions; furthermore, the neural network models used in research are often single models, which cannot effectively demonstrate the superiority of the prediction methods. Therefore, in this study, the research data will be predicted, and the prediction accuracies of different neural network models will be compared and analyzed based on the direct carbon emission data of provincial residents, so as to identify a more suitable neural network model. The chosen model is then combined with ArcGIS for secondary development; specifically, it will be embedded into a direct carbon emission prediction system for Chinese provincial residents, which has great practical application value.

## 2. Literature review

### 2.1 Research on carbon emission prediction

Scholars worldwide have adopted various methods to explore carbon emission prediction. Some scholars use qualitative analysis methods to find out the factors influencing carbon emissions. The IPAT model proposed by Ehrlich et al. is primarily adopted in simulating and predicting carbon emissions, with the general form of “I = PAT,” where I, P, A, and T represent environmental impact, population size, wealth per capita, and technology level factors, respectively; its primary purpose is to evaluate the impact of various factors on carbon emissions quantitatively, and to simulate and predict the development trend of carbon emissions [15]. Greening has utilized the IPAT model to evaluate the impact of the population on carbon emissions in 93 countries over the past 20 years, concluding that the population in developing countries has a more significant impact on carbon emissions than in developed countries [16]. York et al. have utilized a stochastic IPAT model—the STIRPAT model—and time series regression to predict China’s carbon emissions’ peak time under different development trends [17]. Also using the STIRPAT model, Shao et al. have analyzed the factors influencing the carbon emissions in energy consumption in Jiangsu Province, China; and the trends of carbon emissions in different development scenarios were simulated and predicted [18]. By improving the traditional EKC theory based on the endogenous economic growth model—the Moon-Sonn model, Luo et al. predicts the future economic growth pattern of China and the trend of the total carbon emissions generated by China’s future energy consumption, finding that under the current level of technological development, China would reach the peaks of carbon emissions and energy consumptions in 2040 and 2043, respectively [19]. Wang et al. used carbon emission data and Gross Domestic Product (GDP) data in the past 30 years as a benchmark and a prediction model of discrete second-order difference equation to predict carbon emissions and GDP in 2020 [20]. Wang et al. used a Logistic model to simulate the carbon emission of each province in China from 2010 to 2020 [21].

By employing the STIRPAT model, Cui et al. (2018) have analyzed the indicators that could affect carbon emissions’ prediction, with six indicators selected as independent variables, including: population size, urbanization rate, GDP per capita, tertiary industry GDP ratio, energy consumption intensity, and coal consumption ratio. After establishing a prediction model for China’s carbon emissions based on Backpropagation Neural Network (BPNN), they predicted China’s carbon emissions from 2010 to 2015, indicating that China should reduce the GDP growth rate to help realize the carbon emission reduction targets [22]. Ma et al. (2018) proposed the well-known Kaya identity, according to which, the population factor, wealth per capita, energy consumption per unit of GDP and energy intensity are important indicators that affects carbon dioxide emissions in a country or region [23]. Assuming that “carbon emissions were proportional to energy consumption,” Yu et al. (2019) have used the Logistic model to predict the carbon emissions of 30 provinces of China from 2002 to 2020. The outcomes proved that the prediction model’s accuracy was moderate, the prediction value had a high degree of credibility, and the prediction model was systematic, scientific and practical. Therefore, the research results provided effective and scientific methods and reliable data support for formulating future carbon emission reduction policies for China’s various provinces [24]. By summarizing carbon emission prediction models and methods worldwide, Wang et al. (2020) have created a discrete second-order difference equation prediction model to predict China’s carbon emissions in 2020, and the results suggested that China’s carbon emissions’ growth rate would continue to grow in the next ten years and reducing carbon emissions per unit of GDP is of great significance for reducing carbon emissions [25]. After a quantitative analysis of carbon emissions’ driving factors in China, the United States, Japan and Europe, Wang et al. (2020) find that in the past 20 years, the indicators of population and economic development had a very noticeable impact on the hike of China’s carbon emissions, while the indicators of carbon emission intensity and energy intensity had a depressing effect on carbon emissions [26].

### 2.2 Carbon emission prediction by neural networks

Neural network models are also widely adopted in the field of carbon emission prediction. Tian et al. first employed the grey relational analysis method to screen out the factors affecting China’s carbon emissions; then, the BPNN was utilized to predict China’s carbon emissions [27]. Chiroma et al. have also used the BPNN to predict the development trend of China’s carbon emissions from 2010 to 2015 [28]. Acheampong et al. have further utilized the BPNN model to predict China’s coal consumption and carbon emissions, finding that China’s coal consumption and carbon emissions would continue to increase in the next few years, and the growth would be relatively stable [29]. When making predictions on China’s carbon emission intensity based on the combined model of ARIMA and BPNN, Sun et al. find that China’s carbon emission reduction in the next decade would fall behind the expected target, and this conclusion provides a theoretical basis for the designation and adjustment of macroeconomic policies [30].

Zhao et al. (2017) have applied BPNN to predict China’s carbon emissions in 2010 to 2015 based on the STRIPAT model [31]. Hao et al. (2018) put forward a peak prediction model for corporate carbon emission based on gray neural network models to foretell peak corporate carbon emissions, so as to help companies understand their carbon emissions and design carbon emissions reduction paths [32]. Mardani et al. (2019) find that the BPNN model could be used well to predict China’s carbon emissions by following the principle of Grey Relational Analysis (GRA). A holistic, adaptive neuro-fuzzy inference system is proposed to learn to predict and analyze the relationship between renewable energy consumption, economic growth, and carbon dioxide, while forecasting carbon emissions [33]. By adopting a vector autoregressive model, Lin et al. (2019) have analyze the influencing factors of corporate carbon dioxide emissions and determined the principal driving forces of carbon dioxide emissions, finding that based on scenario analyses, the dynamic prediction of China’s PRCI carbon dioxide emissions in 2030 can provide a reference for China to achieve emission reduction targets [34]. Based on actual operating data for iron ore sintering, Ding et al. (2020) have developed an integrated prediction model, which can achieve high-precision prediction of carbon emissions, while providing an effective solution for energy saving and consumption reduction in the actual sintering process. Further, the BPNN model optimized with the Particle Swarm Optimization (PSO) algorithm is utilized to predict the carbon emissions of the heavy chemical industry [35]. Wen et al. (2020) have implemented systematic clustering to screen the world’s carbon emission indicators and used BPNN to forecast the golobal carbon emissions, providing a new carbon emission prediction method [36]. Wu et al. (2021) have applied the direct carbon emissions of the construction industry in Anhui Province in 2008 to 2016 on a sample basis. With the gray prediction model, they also predict the direct carbon emissions of the construction industry in 2017 to 2021 in that province, revealing an uptrend [37].

### 2.3 A summary of previous works

In summary, although both the BPNN and the multiple linear regression model can apply to carbon emission prediction, it is difficult to distinguish in detail their carbon emission prediction accuracy; in addition to the limited carbon emission data, large samples cannot be obtained. To make accurate predictions of carbon emissions, small scales of sample data have become an urgent problem to be solved. On the other hand, the neural network model has proven its substantial application value, with fruitful research results delivered in various fields. With a robust nonlinear relationship observed among the various factors that affect residents’ direct carbon emissions, the neural network model can make good use of its ability to approximate nonlinear functions in predicting the direct living carbon emissions of residents in China’s provinces (municipalities). Although neural network models have already been applied in carbon emission prediction and analysis, they have not been widely used in research on residents’ direct life carbon emissions.

This study selects a relatively easy neural network model, which cannot effectively prove the superiority of the selection of prediction methods. Then, based on studying different neural network models, this study will predict the research data, and compare and analyze the prediction accuracy by considering the direct carbon emission data of provincial residents, purposed to identify a suitable neural network model and combine it with ArcGIS II. Finally, the model will be embedded into a direct carbon emission prediction system for Chinese provincial residents through second development. This approach has great practical value.

## 3. Methods

### 3.1 Calculation methods of carbon emissions

Carbon emissions refer to the average greenhouse gas emissions generated during humans’ production, transportation, use, and recycling of products. Measurements of carbon emissions can be divided into direct and indirect carbon emissions calculation [38]. The direct carbon emission calculation method adopts the carbon emission coefficient method: the carbon emissions in residents’ lives are measured based on statistical data on various fossil energy sources. Here, in reference to the calculation method of international greenhouse gas emissions [39], the measurement is as follows:

$$C_{\text{ij}}=E_{\text{ij}}\cdot EF_{\text{j}}$$ (1)

where i refers to the i-th region, and j refers to the j-th fuel. When j is 1, 2, 3…19, it represents 19 types of energy sources such as coal, oil, natural gas, heat, electricity and etc. *C*_ij_ is the carbon emission of the j-th fuel in the i-th region, *E*_ij_ is the final consumption of the j-th fuel in the i-th region, and *EF*_i_ is the CO_2_ emission coefficient of the j-th fuel. This method has to compute massive residential energy consumption data, which is a time-consuming process.

Indirect carbon emission calculation refers to judging the consumption of energy by residents in their daily lives to calculate the carbon emissions, which corresponds to residents’ indirect energy consumption. The commonly used models include the input-output model, life cycle assessment (LCA) method, hybrid life cycle method, and consumer lifestyle method [28]. The equation of the input-output model is as follows:

$$CF=F\times E_{\text{j}}=F\times D\text{j}\times {\left(I-A\right)}^{\text{−}1}$$ (2)

where CF represents indirect carbon emissions, F represents the living consumption of residents, E_j_ represents the indirect carbon emission intensity of energy consumption in sector j, D_j_ represents the direct carbon emission intensity of energy consumption in sector j, A represents the matrix of the input and output direct consumption coefficients, and I represents the identity matrix of the same order of A. However, the scope of this model is broad, and it covers more sectors. As a result, it entails an extended period of preparation before calculation, with many significant limitations.

The LCA method is mainly used to evaluates the processing, manufacturing, transportation and sales of a commodity in its entirety. The commodity’s life cycle inventory must be established before the LCA method is deployed. Compared with the IO model, the LCA method requires more workforce, material resources, and workload. Since the focus of this study is to provide a prediction method and operating platform for exploring the development trend of carbon emissions, the direct carbon emissions of residents in the provinces with relatively simple accounting methods and relatively little workload are chosen as the data basis.

### 3.2 Prediction of carbon emissions prediction based on the BPNN model

**Fig 1:**
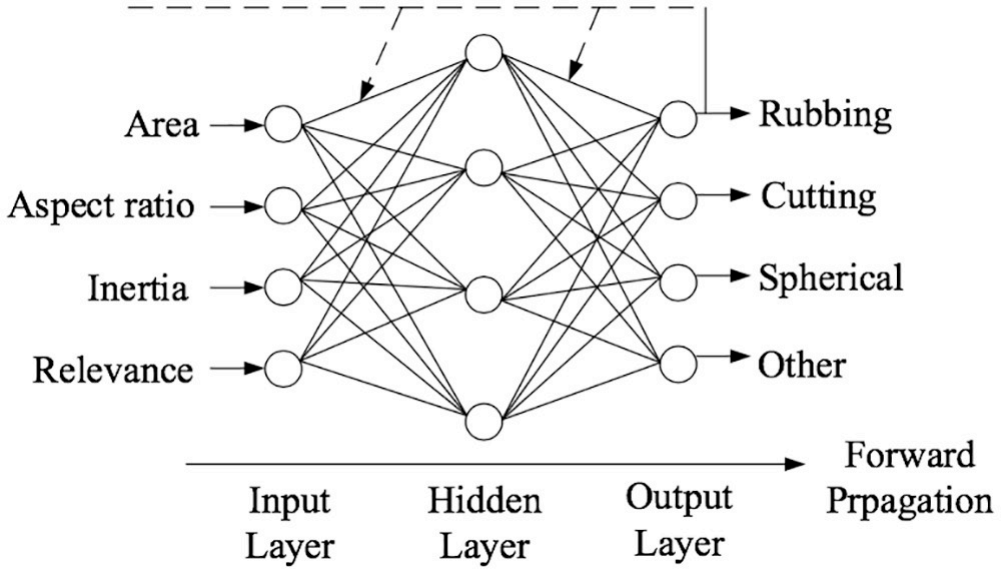
Schematic diagram of the BPNN structure.

As a supervised learning algorithm, the BPNN consists of an input layer, a hidden layer and an output layer, with a full connection formed between neurons at various layers [40]. The learning process is divided into forwarding propagation and backpropagation: The former goes from the input layer to the hidden layer and the output layer; and the latter is for signals to propagate forward from the output layer. A weight value limits each layer of the transfer. The data information is processed by combination with the neuron activation function, hidden layer neuron quantity, and weight adjustment rules. This model can deliver different network functions, and its specific structure is shown in Fig 1.

Based on the direct carbon emission data processed for Chinese provinces, the per capita carbon emission of Fuzhou City is selected as the testing data for the neural network. The specific process is as follows: (1) Construct the training samples. All data are divided into 20 data samples. Since 1999, the last data every five years are the algorithm’s input value. The first 18 samples will be used as the training sets, and the last two as the testing sets. And (2) make the carbon emission prediction and result output. For the trained network, the Fuzhou carbon emission data in 2015–2019 are predicted, and the results will be used as output. The residual error, relative error and MSE are selected as the comparison standard for the prediction accuracy.

### 3.3 Carbon emissions prediction based on RBF neural network model

**Fig 2:**
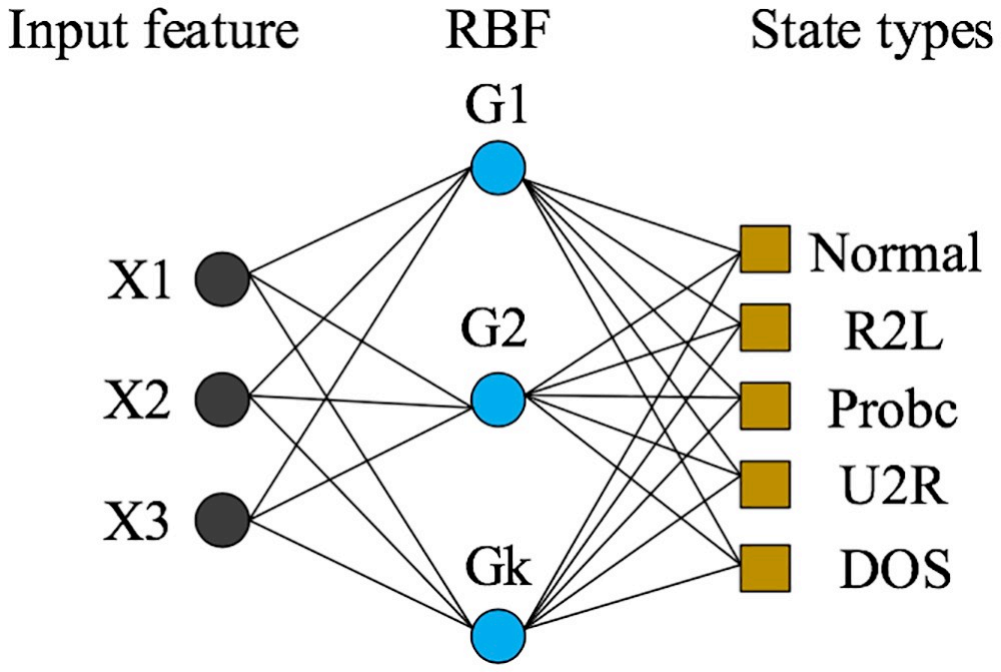
Schematic diagram of the RBF neural network structure.

The Radial Basis Function (RBF) neural network is a three-layer feedforward neural network model with only one hidden layer, whose input layer uses a non-weighted connection to directly transfer the data to the hidden layer’s neural unit [41]. As shown in Fig 2 for the structure of the RBF neural network model, it uses radial basis functions as the basis functions. The hidden layer is used to map the input data to the hidden layer space, so as to complete the data’s nonlinear transformation. The output layer is often linearly transformed.

Based on the direct carbon emission data processed for Chinese provinces, the per capita carbon emission of Fuzhou City is selected as the prediction purpose of the neural network. The specific process is as follows: (1) Sample data structure: The data are based on a 20x31 matrix. The data from other provinces except Fuzhou are used as the learning samples, while Beijing’s data are used for testing. And (2) the new rb function is used to set network parameters, such as allowable error, diffusion factor, and neurons. The same evaluation indicators as for the BPNN are adopted to output the results.

### 3.4 Carbon emission prediction based on Elman neural network model

**Fig 3:**
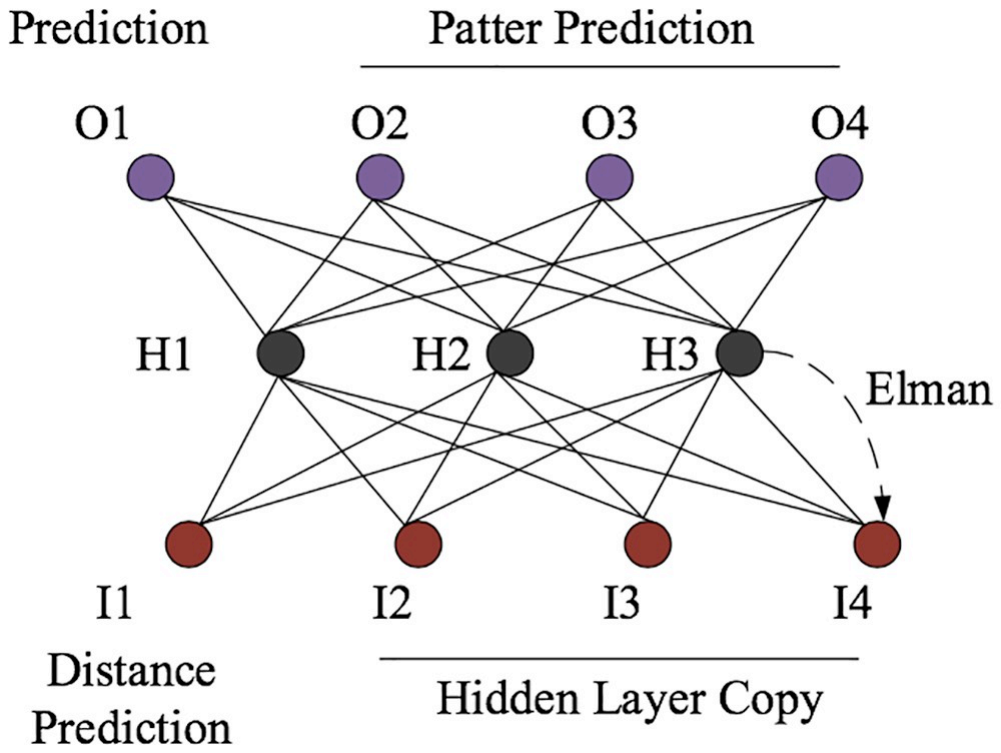
Schematic diagram of the Elman neural network structure.

Elman neural network is a dynamic feedback neural network, or a neural network model with local memory and feedback capabilities. This model can deliver good convergence speed and high prediction accuracy thus adopted in many fields [42]. Compared with the other network models, it provides an additional undertaking layer with memory and feedback functions, with its specific structure shown in Fig 3. The undertaking layer can give feedback to the hidden layer through data and realize dynamic memory and feedback functions.

Based on the direct carbon emission data processed for Chinese provinces, the per capita carbon emission of Fuzhou City is selected as the prediction purpose of the neural network. The specific process is as follows: (1) Training sample determination: Consistent with the BPNN’s training sample method, the rolling prediction is realized by using a time series. (2) The Elman net function in the Matlab neural network toolbox is used to establish the Elman neural network. Besides, the delay layer, hidden layer neuron size, training function, and other network parameters are set. And (3) this is consistent with the time selection and measurement indicator of the BPNN, and the results are outputted finally.

### 3.5 Carbon emission prediction based on the GRNN neural network model

**Fig 4:**
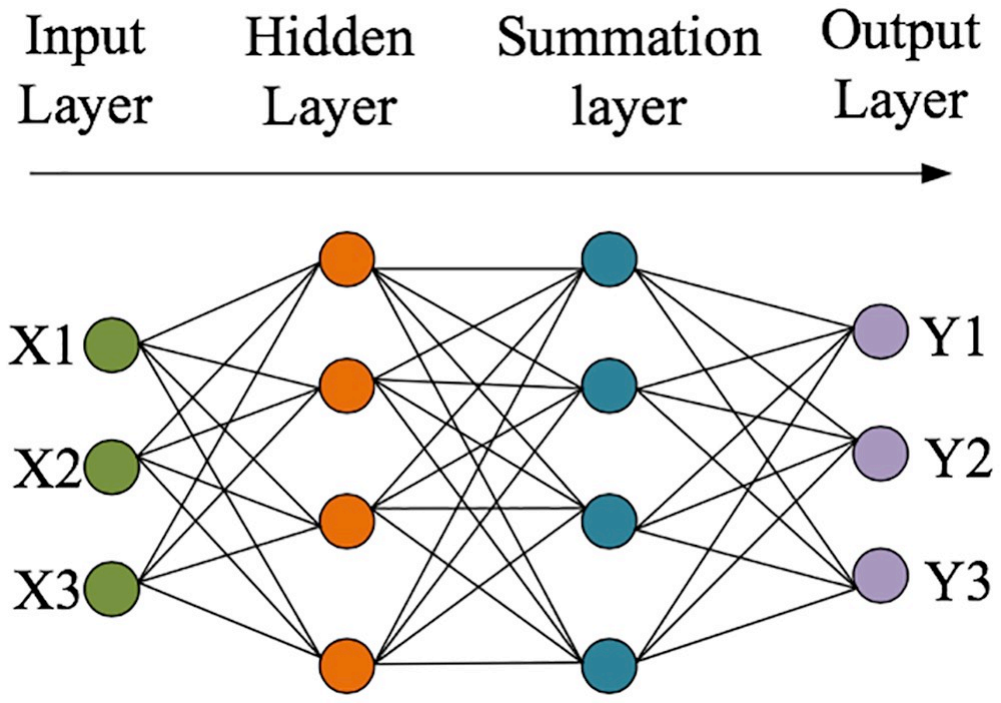
Schematic diagram of the GRNN neural network.

The Generalized Regression Neural Network (GRNN) is a neural network algorithm with radial basis functions. Due to its strong curve mapping ability, flexible network structure, high fault tolerance, and fast learning speed, it has been applied in the construction of multiple network models [43]. The neural network can provide a better prediction effect under the premise of a few samples. Its specific structure is shown in Fig 4. In this study, 90% of the residential carbon emission data in Fuzhou are used as the training sample, and the rest as the testing sample. The measurement indicators and result outputs are similar to those for the BPNN.

### 3.6 System requirements and implementation

#### (1) System requirements

The system should have the essential functions of GIS software for implementing basic map operations. In terms of parameters, it is necessary to call the Matlab software, modify the relevant parameters of the Elman neural network, designate the to-be-predicted provinces and years, and display and save the carbon emission prediction results. In terms of display, thematic map creation functions are added to the system, including pie charts, bar charts, and graded coloring charts, so as to facilitate more intuitive understanding of the carbon emissions in different years and provinces as well as the composition of various energy consumption types. Additionally, it has other necessary functions for the production of related maps.

#### (2) System implementation

This system is developed in C#.NET environment based on ArcGIS Engine 10.2, combined with the neural network toolbox in Matlab R2014a. As a language derived from C and C++, although C# inherits the powerful programming functions of C and C++ languages, it eliminates the latter two’s complex features. ArcGIS Engine is a complete set of embedded GIS component libraries and tool libraries that package ArcObjects. It provides a complete class used by developers to build custom applications. The neural network toolbox in Matlab offers a practical technical method for predicting China’s provincial carbon emissions. In this development environment, the system calls the Elman neural network prediction model realized by using Matlab neural network toolbox in the form of the.dll format, thereby delivering the integration of system functions.

#### (3) Data sources

Residents’ energy consumption structure varies, and the types of energy consumption in different regions and by different households are also quite different. Most of the calculation methods for carbon emissions use provinces and cities as the administrative unit [44]. Carbon emission coefficients can measure the direct carbon emissions of per capita residents in China’s 30 provinces, municipalities, and autonomous regions from 1999 to 2019. In particular, data of 19 residential energy consumption types come from the 2000–2019 *China Energy Statistical Yearbook* (https://data.cnki.net/Trade/yearbook/single/N2017110016?z=Z024); the CO_2_ emission coefficients can be accessed at *IPCC National Greenhouse Gas Emissions Inventory Guidelines 2006* (https://www.ipcc-nggip.iges.or.jp/public/2006gl/chinese/index.html); the electrical carbon emission coefficients of all provinces and municipalities are derived from the 2019 *China Regional Power Grid Baseline Emission Factors* (https://www.mee.gov.cn/ywgz/ydqhbh/wsqtkz/202012/W020201229610353340851.pdf); data of the total, urban, and rural populations refer to the 2000–2019 *China Statistical Yearbook* (http://www.stats.gov.cn/tjsj/ndsj/). What should be emphasized is that due to the lack of relevant statistical data, the study area does not include the Tibet Autonomous Region, Taiwan Province, Hong Kong, and Macau Special Administrative Region. Besides, the data of Ningxia Hui Autonomous Region in 2000 to 2002 are missing, so they are complemented by using the moving average method. Mean Squared Error (MSE) refers to the expected value of the square of the difference between the parameter estimate and the parameter value. As a convenient method to measure the “mean error,” MSE can evaluate the degree of data’s changes. The smaller the value of MSE, the better the prediction model’s accuracy to describe the experimental data [45].

#### (4) Model training

The training structure plays a vital role for network prediction’s accuracy. The model chooses the rolling prediction output method on a time series to predict the direct carbon emissions of residents in Fuzhou based on multiple experiments, so as to improve the network convergence performance and prediction accuracy. The 20-year carbon emission data are divided into 16 data samples; in other words, the carbon emission data of every four consecutive years after 1999 are used as the data input, and the carbon emission data of the fifth year are used as the data output. For example, the carbon emission data in 2016, 2017, 2018 and 2019 are used as input, and those in 1999 are as output. Hence, 16 pairs of data samples are constructed, with the first 14 pairs of sample data used as training samples and the last 2 pairs as test samples, so as to evaluate the prediction accuracy of the BPNN.

## 4. Results and discussion

### 4.1 Total carbon emissions of residents of different provinces in different years

**Fig 5:**
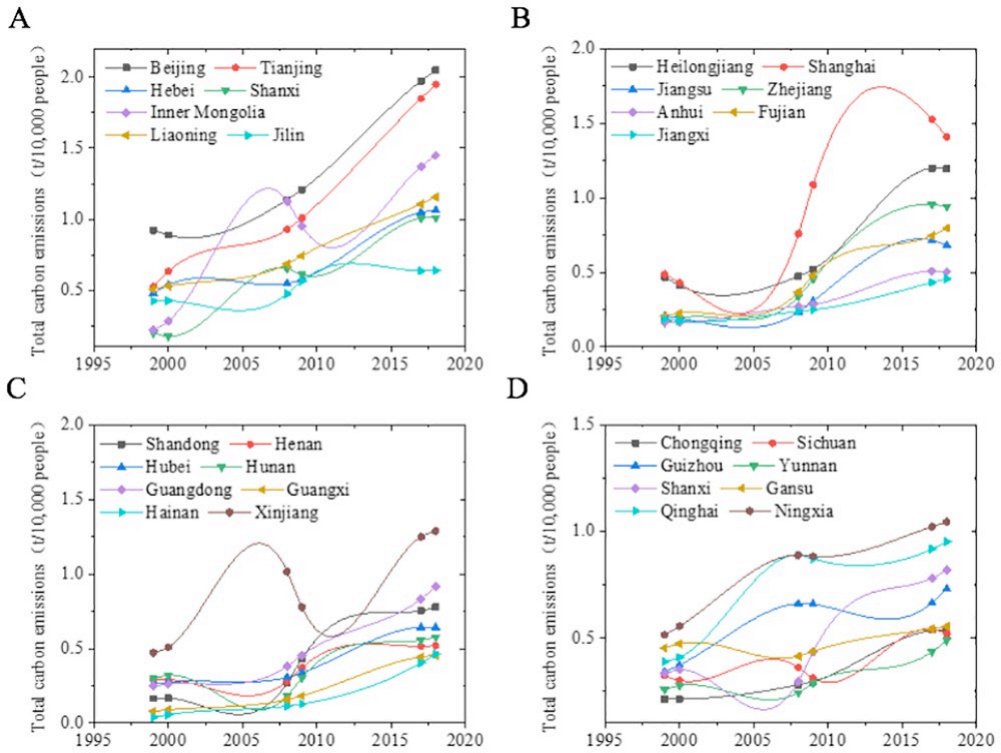
Total carbon emissions in different years in different provinces.

**Fig 6:**
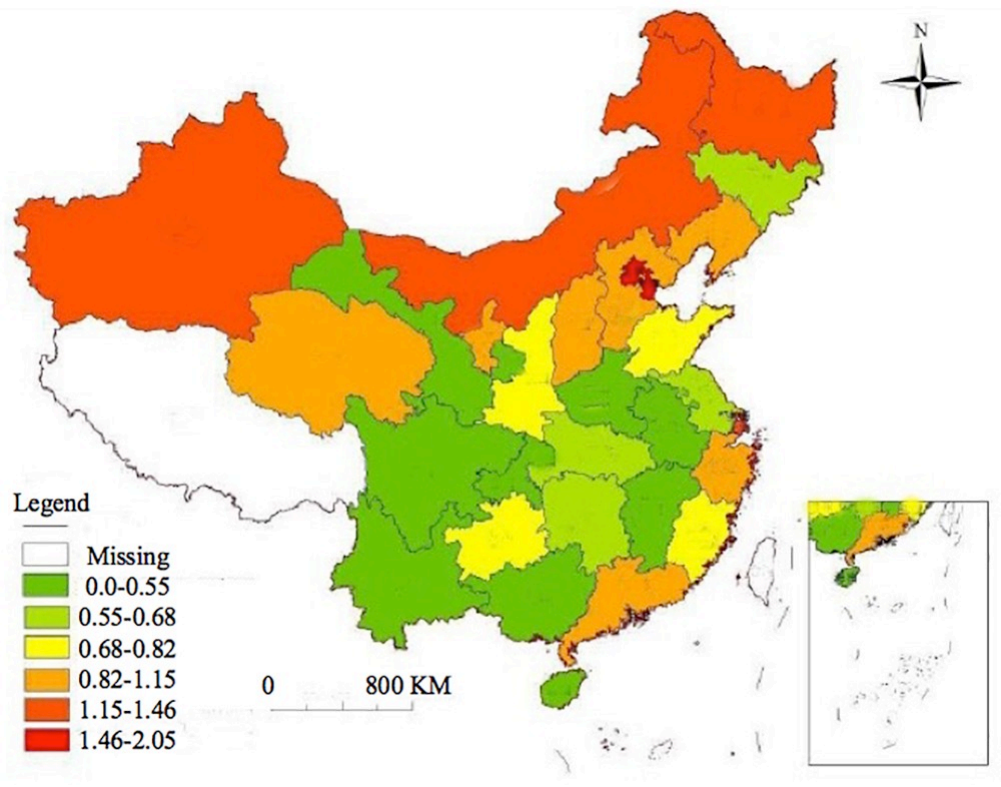
Colored map of carbon emission classification in different provinces in different years. Republished from “Prediction of Direct Carbon Emissions of Chinese Provinces using Artificial Neural Networks” under a CC BY license, with permission from ArcGIS Engine10.2, original copyright 2020.10.22.

The total carbon emissions of residents shown in Fig 5 are obtained based on different provinces’ data in different years. The total residential carbon emissions of Beijing, Tianjin Municipality, Hebei Province, Shanxi Province, Inner Mongolia Autonomous Region, Liaoning Province and Jilin Province in Northern China had not grown much from 2000 to 2010. However, the growth rate has risen sharply since 2010. In Tianjin, the total residential carbon emissions in 2018 were increased by 4 times compared with those in 2010. Although the total carbon emissions of the five provinces of Jiangsu, Zhejiang, Anhui, Fujian and Jiangxi in Southern China have increased, the growth rate has slowed down significantly. Shanghai Municipality and Shandong Province have shown large fluctuations, i.e., first increasing and then decreasing. Such fluctuations may be closely related to local environmental protection policies. In provinces of Shanxi, Gansu, Ningxia, Qingdao and Guizhou in north-western China, the residential carbon emissions have increased briefly first, then decreased, and later increased again slowly. The results above demonstrate that the northern China’s residents’ carbon emissions are significantly higher than those of southern residents. A possible reason is that although the population in southern China is more dense, the greening and environment conditins are good there; furthermore, most of the heavy industrial areas are concentrated in northern cities, and there are more factories in light industry and electronic information industry in the southern cities. Fig 6 shows the carbon emission classification for different provinces in different years.

### 4.2 Prediction results of different carbon emission prediction models for different provinces

**Fig 7:**
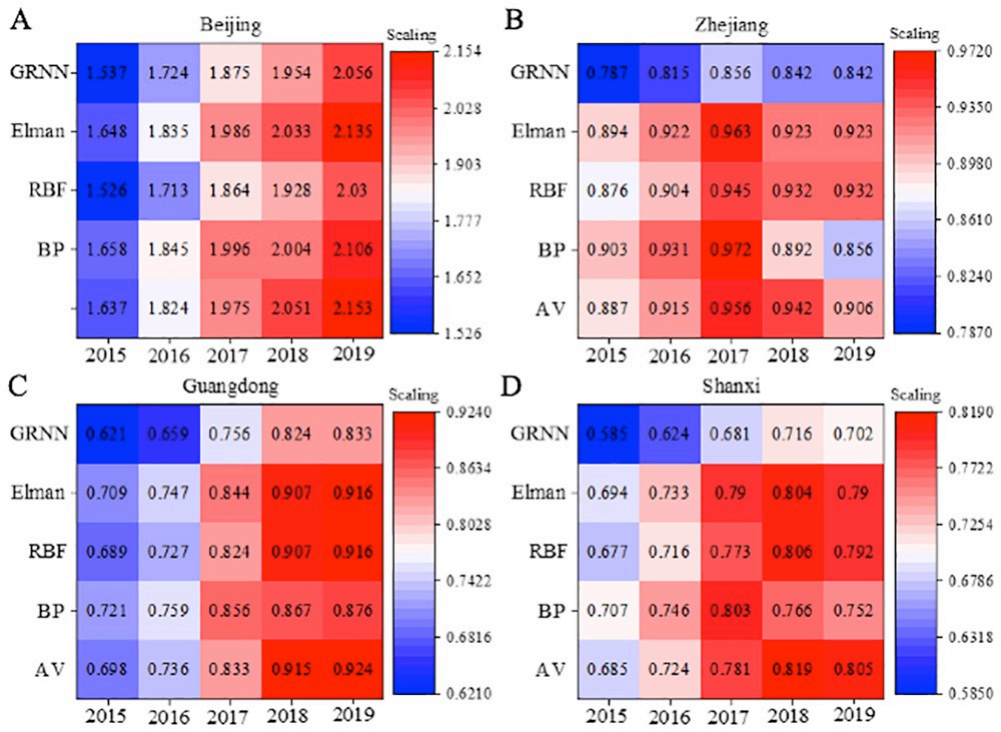
Prediction results of different carbon emission prediction models in different provinces. Republished from “Prediction of Direct Carbon Emissions of Chinese Provinces using Artificial Neural Networks” under a CC BY license, with permission from ArcGIS Engine10.2, original copyright 2020.10.22.

Fig 7 presents the prediction results of different carbon emissions for different provinces. Four geological areas, namely Beijing (representing the Northeast China), Zhejiang Province (representing the East China), Guangdong Province (representing the South China), and Shaanxi Province (representing the West China). In this figure, the abscissa represents different years, and the ordinate represents different forecasting models.

Besides errors in 2018 and 2019 predicted values by GRNN, all other neural networks can make predictions on future carbon emission trends that are consistent with the actual changes. The prediction results are satisfactory. Carbon emissions predicted by these three neural networks are consistent with the actual values, similar to the conclusions drawn by Tang et al. (2020) [46]. Nevertheless, RBF made large errors in the 2018 prediction. Other prediction results are satisfactory. Compared with BPNN, RBF has simpler structure and does not require repetitive training. However, results in Fig 7 reveal its low prediction accuracy and huge prediction errors. The same conclusions have been proved by Yang et al. (2020) [47] in their work. BPNN and Elman neural network can provide accurate predictions on carbon emissions in Beijing, whose errors are lower than 0.015, proving the excellent performances of both models. Moreover, Elman neural network outperforms BPNN and RBF regarding predictions on all four areas. Theoretically, they can predict all carbon emission data covered. To achieve better performance, BPNN requires multiple training referring to experience. However, huge errors may occur during training. Also, BPNN is less robust and more random than Elman neural network, which has been proved by Zhang et al. (2018) [48]. To sum up, Elman neural network can provide outstanding performance in carbon emission prediction, more suitable to solve the problems in the present work.

### 4.3 Performance evaluation of different carbon emission prediction models

The above data are tested with Standard Error (SE) and Relative Error (RE) analyses, with the obtained shown in Table 1. For different years, compared with RBF neural networks, the BPNN needs to be trained for many times based on experiences before it can be determined as the optimal network; hence, a significant prediction error exists in the training process. The RBF neural network model is less robust and more random than the Elman neural network, so the prediction accuracy of the former is slightly inferior to the latter. The changing trends of carbon emission predictions delivered by the three kinds of neural networks are basically consistent with the actual carbon emissions. Except for some large errors with the predictions for 2017 as obtained by using the RBF neural network, the remaining prediction results can meet the expectations. Compared with the BPNN model, the RBF neural network model has a simple structure, without requiring repeated trainings, while converging quickly. However, with low prediction accuracy and large errors, it is unsuitable for predicting the data. The BPNN model and the Elman neural network model can both deliver high accuracy in predicting carbon emissions in Beijing, with errors below 0.015, and both can give good prediction results. Thus, they can theoretically be used to predict carbon emission data. In addition, the BPNN model requires multiple trainings based on experiences; however, large prediction errors may occur occasionally during the training process. Compared with the Elman neural network, the BPNN has insufficient robustness and greater randomness, and it is slightly inferior to the former in terms of convergence speed and prediction accuracy. In a word, the Elman neural network has superior performance in carbon emission prediction and is more suitable for the issue in this study, because the above results demonstrate that the Elman prediction model has the best effect.

**Table 1:** Performance evaluation of different carbon emission prediction models.

Networks	2016		2017		2018		2019	
	SE	RE (10^−2^)	SE	RE (10^−2^)	SE	RE (10^−2^)	SE	RE (10^−2^)
BPNN	0.0128	0.6479	-0.0095	-0.4638	0.0138	0.486	0.245	-0.539
RBF	-0.01	-0.5063	-0.1028	-0.5338	-0.011	-0.337	-0.124	-0.375
Elman	0.0042	0.2124	-0.0149	-0.0121	0.0035	0.115	-0.011	-0.198
GRNN	-0.0138	-0.586	-0.114	0.376	-0.0167	-0.358	0.187	-0.449

**Fig 8:**
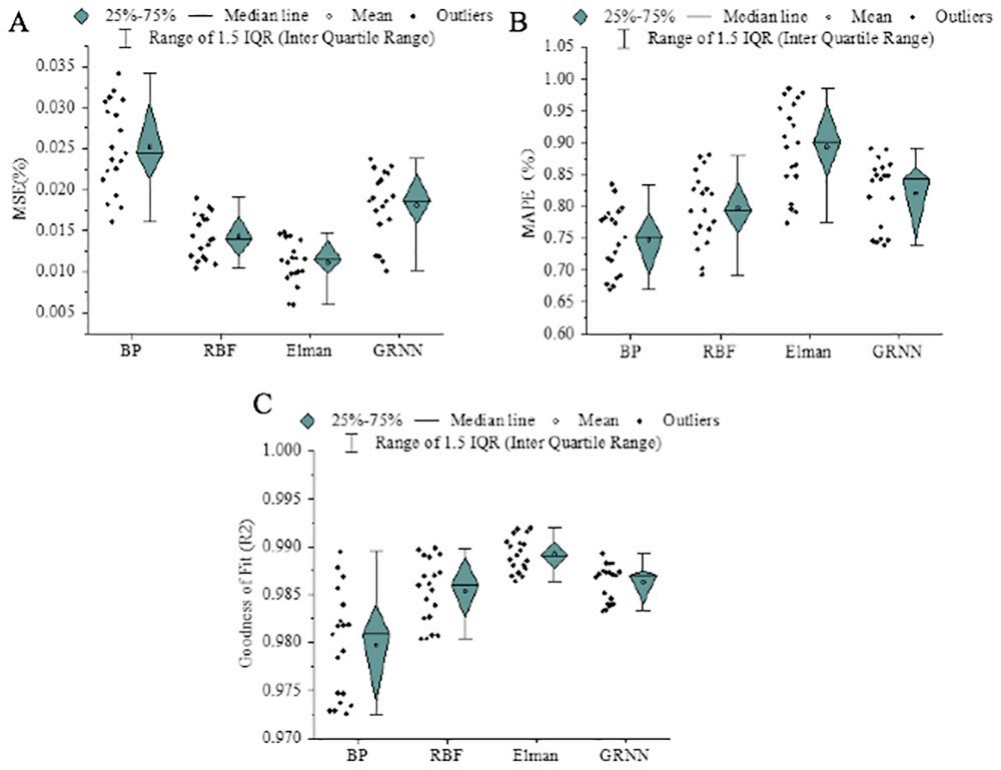
Prediction performance evaluation of different carbon emission models.

In Fig 8, the abscissa denotes different model methods, the ordinate describes different indicator results. Fig 8A–8C displays the results of Mean Square Error (MSE), Mean Absolute Percentage Error (MAPE), and Coefficient of Determination (R^2^), respectively. The smaller the MSE, the more accurate the model. Specifically, BPNN provides an MSE of 0.252 after deep learning, the worst compared with other neural networks; in contrast, Elman neural network provides the best accuracy, 55.93% higher than BPNN. The larger the MAPE, the better the prediction performance. Similar to the results of MSE, BPNN is the worst while Elman performs the best; the latter makes an improvement of 19.48% than the former. However, regarding R^2^, Elman is not much different from other neural networks. Shen et al. (2019) analyzed and predicted NH_3_ concentration in atmosphere. They found that Elman performed much better in accuracy than BPNN [49]. Hu et al. (2019) predicted China’s carbon emissions using the Firefly Algorithm-optimized Elman neural network. They discovered that Elman could provide more excellent performance than GRNN [50].

The larger the average MSE, the better the model’s prediction performance; this is consistent with the accuracy results. Compared with the BPNN model, the Elman network model’s prediction performance is improved by 19.48%. In terms of model fit, the Elman network model is not much better than other network models. The above results are consistent with the findings of Zheng et al. (2021) [51].

### 4.4 Prediction results of direct carbon emissions from Chinese residents in the future

Based on the above model comparison, the Elman carbon emission prediction model is selected to predict Chinese residents’ direct carbon emissions from 2020 to 2035, with the results shown in Table 2. without considering other influencing factors (especially policies), Chinese residents’ direct carbon emissions in the next 15 years will still demonstrate a steady growth trend in the next five years. After 2027, direct carbon emissions per capita will show a slight downward trend. Besides, in 2032, the carbon emissions per capita will reach 0.8882 t/10,000 people. After reaching a weak peak, it will experience a slight decrease. The above results show that, theoretically, the peak of carbon emissions is expected to appear in around 2025–2030.

**Table 2:** Prediction results of Chinese residents’ direct carbon emissions from 2020 to 2035.

Scenes	Carbon emissions (t/10,000 people)				
2020–2025	0.8829	0.8831	0.8855	0.8852	0.8873
2025–2030	0.8882	0.8879	0.8882	0.8874	0.8876
2030–2035	0.8879	0.8880	0.8883	0.8881	0.8879

## 5. Conclusion

In this study, residents’ direct carbon emission data in various provinces and cities in the past 20 years are sorted out and calculated. Based on the nonlinear features of the carbon emission data, the BPNN, RBF and Elman neural network models are chosen. A particular city’s carbon emission data are used as the test data to make predictions, so as to verify the neural network model’s feasibility for carbon emission prediction. The network with better performance is determined by comparison of the prediction performance; then, it is embedded in a direct carbon emission prediction system for Chinese provincial residents, so as to provide an effective operating platform for carbon emission analysis and prediction.

The results demonstrate that the three neural network models are effective in predicting carbon emission data and can provide practical and feasible operating methods for carbon emission prediction and analysis. The prediction results for 2014 show large errors, and the long-distance prediction results cannot be obtained very accurately. Both BPNN and Elman neural networks can deliver high prediction accuracy, but their MSE values are slightly different. Theoretically, BPNN and Elman neural networks can be used to predict carbon emission data. However, in comparison, the BPNN model is slightly cumbersome in the process of construction and training, and its robustness and fault tolerance are slightly lower than those of Elman neural network. Therefore, after a comprehensive comparison, the Elman neural network model is chosen to predict carbon emission data.

In the absence of external forces, and with the free development of carbon emissions, Chinese residents’ direct carbon emissions will continue an upward trend at a fast-to-slow growth rate in the next few years. This trend will gradually become flat after 2020, while the direct carbon emission per capita will reach its peak in 2032. Although the advantages and disadvantages of different models have been analyzed as much deeply as possible, the following shortcomings exist due to objective limitations: (1) the data on direct carbon emission of residents in each year and each province will inevitably produce errors under the influences of such factors as the availability of statistical data over the years and ever-changing statistical calibers, leading to possible deviations in the conclusions derived; and (2) at present, an ideal network model is often obtained by empirical methods. However, the network testing process is somehow cumbersome, with huge workload required. In this process, subjective factors will impact network performance. In the future, these two weaknesses will be explored more profoundly, so as to predict and analyze residential carbon emissions accurately, quickly and efficiently.

## Supporting information

S1 Data (XLSX)

S2 Data (RAR)
